# Mometasone furoate is not superior to saline for chronic rhinitis in the elderly

**DOI:** 10.1016/j.waojou.2019.100064

**Published:** 2019-10-14

**Authors:** Victor Carvalho, Beni Olej, José Rodrigo de Moraes, Jose Laerte Boechat

**Affiliations:** a Unidade de Pesquisa Clínica, Hospital Universitário Antônio Pedro, Universidade Federal Fluminense. Rua Marquês do Paraná, 303, 4º andar, Niterói, 24033-900, Rio de Janeiro, Brazil; b Instituto de Matemática e Estatística, Universidade Federal Fluminense. Rua Professor Marcos Waldemar de Freitas Reis, Campus do Gragoatá, Niterói, Rio de Janeiro, Brazil; c Departamento de Alergia e Imunologia, Faculdade de Medicina, Universidade Federal Fluminense. Rua Marquês do Paraná, 303, 2º andar, Niterói, Rio de Janeiro, Brazil

**Keywords:** Aged, Rhinitis, Mometasone furoate, Immunosenescence, Peak nasal inspiratory flow, PNIF, peak-nasal inspiratory flow, VAS, visual analogic scale, SNOT-22, sinonasal outcome test, AR, allergic rhinitis, NAR, nonallergic rhinitis, CE, corticosteroids

## Abstract

**Introduction:**

Prevalence of diseases associated with ageing is rising; among these are the rhinologic problems. Chronic rhinitis appears as one of the most common worrisome nasal disorders in this age group. At the same time, the allergic form diminishes because of the immunosenescence.

**Objective:**

This study aimed to evaluate the effect of a corticosteroid nasal spray (mometasone furoate) over nasal patency and the severity of rhinitis and its impacts on quality of life as compared with the saline nasal spray.

**Methods:**

This open label-trial randomized subjects ≥60y with chronic rhinitis (allergic and nonallergic rhinitis) with mometasone spray 100mcg/d and isotonic saline nasal spray or saline alone for two weeks. The primary endpoint was the improvement in nasal patency evaluated by the peak nasal inspiratory flow (PNIF). Secondary outcomes included the severity of symptoms and the quality of life assessed by a visual analogic scale (VAS) and the sinonasal outcome test (SNOT-22), respectively.

**Results:**

Forty patients underwent randomization, in equal number in each group of treatment, either with allergic (AR) and nonallergic rhinitis (NAR). At week 2, the mean PNIF score was 79.5 in the corticosteroid (CE) plus saline group and 82.0 in the saline group (p = 0.37). Also, SNOT-22 and VAS were not improved with the addition of mometasone furoate.

**Conclusions:**

Treatment with mometasone furoate nasal spray plus isotonic saline is not superior to saline alone in elderly patients with rhinitis in respect of improving nasal patency, quality of life, and reducing the intensity of symptoms.

**Trial registration:**

The trial is registered at the Brazilian Clinical Trials Registry (ReBEC) #RBR-498bnq. Registered 05 July 2017.

## Background

According to the United Nations, up to the year 2050, the population of those who are over 60 years will pass from the current number of 900 million to 2 billion.^1^ Because of this, the prevalence of diseases associated with ageing is rising. That presents a challenge to the public health systems around the world. The rhinologic problems are among the most common geriatric disorders in daily practice,^2^ and they negatively affect quality of life.^3^

Rhinitis is the inflammation of the nasal mucous membrane. The main symptoms are rhinorrhea, nasal blockage, sneezing and itchy nose. The non-infectious chronic rhinitis can be divided into two groups: allergic rhinitis, whose symptoms are the result of IgE-mediated inflammatory response, and nonallergic rhinitis, non-IgE mediated response. More than 60% of rhinitis in patients with more than 50 years is nonallergic rhinitis.^4^ This can be explained by the decline of the immune system with age, a term known as immunosenescence, which involves profound changes in the function of immune T and B cells.^5^ There is also the presence of age-related nasal changes that contribute to the exacerbations and the severity of rhinitis, including the weakening of connective tissue at the lateral and septal cartilages, the increase of the cholinergic activity, the reduction of mucosal blood flow and an impaired mucociliary function. These changes lead ultimately to modifications in the length of nasal cavity, dryness and irritation of nasal mucosa and excessively thick mucus in older adults.^6^

One of the cornerstones in the management of rhinitis is environmental control. However, when the symptoms are more severe and persistent, nasal corticosteroids are the treatment of choice.^7^ Although they are well tolerated in the elderly,^5^ there are few studies and research in the treatment of rhinitis in this age group. Consequently, we conducted a comparative trial to evaluate the efficacy of mometasone furoate, a corticosteroid nasal spray, in geriatric patients with chronic rhinitis in addition to saline spray versus saline alone through subjective assessments of severity and quality of life and its effect over the nasal patency, which is in turn an objective measure of the severity of rhinitis.

## Methods

### Trial design and population

In this single-center study, we compared mometasone furoate plus isotonic saline nasal spray with saline alone. This is a randomized, open-label and active comparator trial that recruited volunteered patients with age equal to or above 60 years old with chronic rhinitis seen in an outpatient allergy clinic in Rio de Janeiro, Brazil. Participants were included after they fulfilled the inclusion criteria: at least 2 chronic symptoms of rhinitis, (congestion, rhinorrhea, itching of the nose or sneezing) for at least 1 hour daily and 2 consecutive weeks per month. Those with primary or secondary immunodeficiency, mechanical obstruction of upper airways and respiratory infection in the last 2 weeks were excluded from the trial. The trial was conducted by following ethical principles of the Declaration of Helsinki and per under the Consolidated Standards of Reporting Trials guidelines.

### Intervention

Participants were not allowed to take any allergic treatment (systemic and topic anti-histaminic and corticosteroids) for 2 weeks. Clinical and laboratory data were obtained with clinical examination, skin prick test and blood samples for total IgE (reference range 1–200 Ku/L) and peripheral blood eosinophils. For another 2 weeks, all the volunteers used isotonic saline nasal spray 4 times a day. In the same period, 1 group used in association mometasone furoate nasal spray 2 doses of 100mcg/d, 1 time a day.

### Randomization

The randomization scheme was generated using a computer-generated code. At first, patients were separated into 2 groups according to the results of the prick test: allergic and nonallergic. After that, in times apart, the participants of each group were randomly allocated in arms of intervention so that both arms of treatment have a proportional number of allergic and nonallergic patients. The trial investigators enrolled and assigned the participants to interventions.

### Outcomes and assessments

The primary outcome was a change in the peak nasal inspiratory flow (PNIF), to assess nasal patency objectively. The significant clinical difference was of 30 L/min. Secondary outcomes were changes in the combined nasal symptoms intensity score rated by a 10-point visual analogue scale (VAS) at 2 weeks, with ‘0’ indicating no symptom and ‘10’ the worst possible discomfort and in the SNOT-22 (sinonasal outcome test) questionnaire of quality of life. SNOT-22 is the only nasal chronic problem questionnaire of quality of life that is validated to Brazilian Portuguese.^8^ This consists of 22 questions scored between 0 and 5, with higher scores meaning more significant problems.

Before the intervention, nasal symptoms were rated by VAS and patients answered the SNOT-22 questionnaire. At the same time, it was measured the PNIF using an In-Check portable peak flow meter (Clement Clark International, Harlow, Essex, UK). Three satisfactory maximal inspirations were obtained, and the highest of the 3 results was taken as the PNIF. At week 2, SNOT-22, VAS and PNIF were repeated in all participants. Data of adverse events were also collected.

### Sample size and statistical analysis

Initially, a pilot sample of 15 patients per group was used for sample size determination. A minimum sample of 40 patients (20 per group) was determined to provide the clinical trial with a power of 80% to detect a substantial difference of 30 L/min (significance level of 5%) in the PNIF score at week 2.^9^^,^^10^ For secondary outcomes, it was considered differences of 15 points in SNOT-22 and 2 points in the 10-point combined nasal symptoms VAS.^8^^,^^9^

The distributions of baseline demographic and epidemiologic characteristics of patients randomly allocated in the 2 groups of treatment were compared with the Chi-squared test. The SNOT-22, VAS and PNIF scores were compared between 2 groups of intervention at baseline and after the treatment using Mann-Whitney test.

Additionally, these scores were compared in a subgroup analysis, considering certain individual strata (presence of atopy, asthma and elevated total IgE). For both tests, significant statistic differences were considered at a significance level of 5% (p-value ≤0, 05). Analyses were performed with the use of SPSS software for Windows, version 20.0.

## Results

### Demographic data

**Fig. 1:**
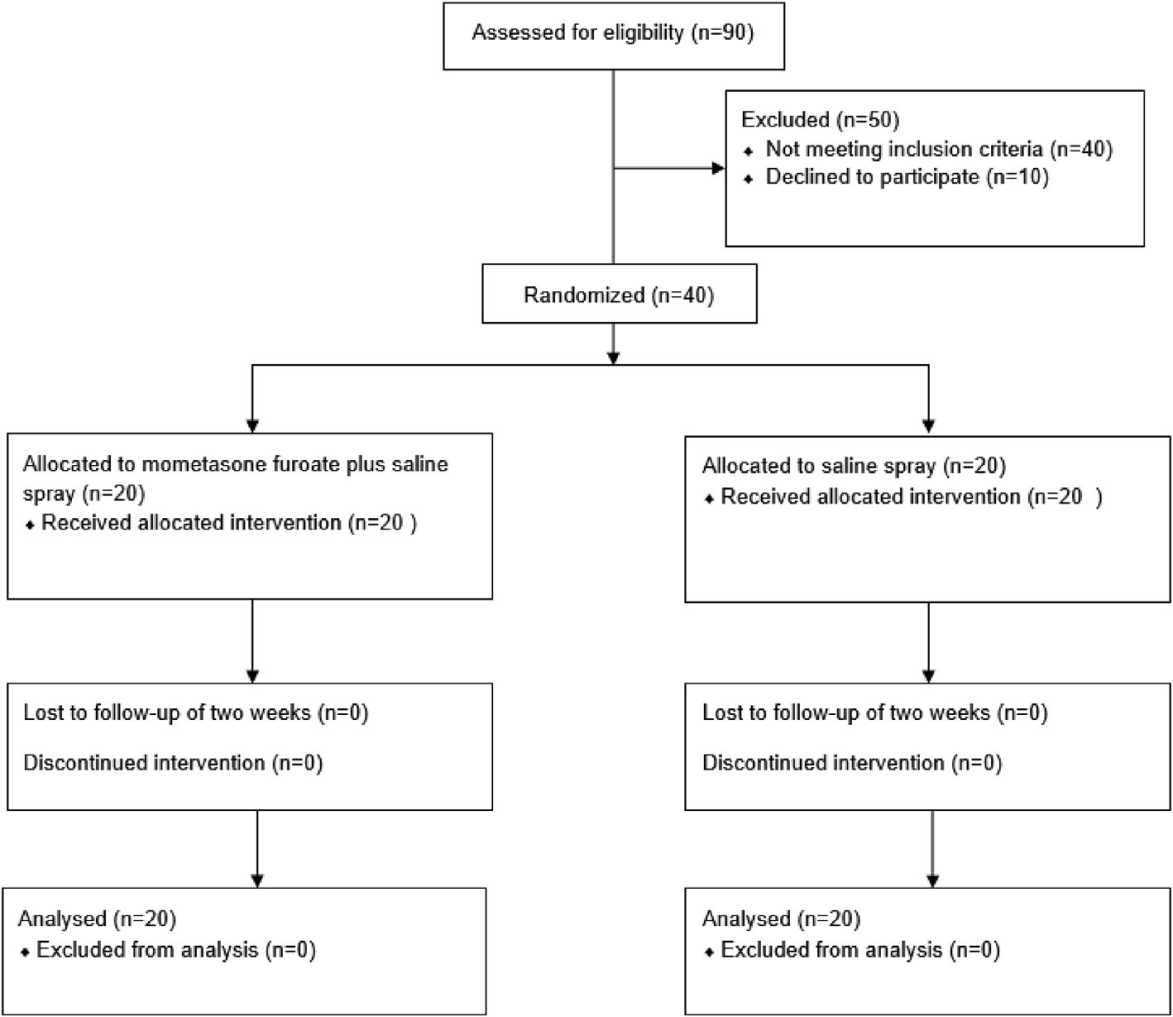
Trial flow chart

**Fig. 2:**
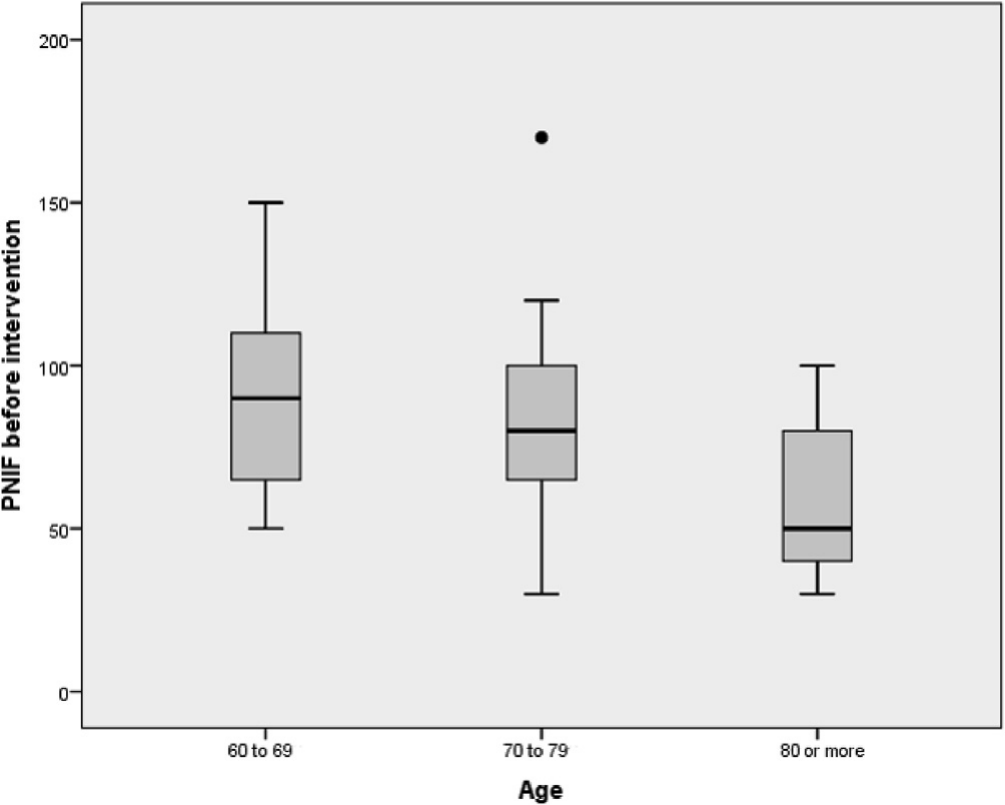
Boxplots of the baseline values of PNIF distributed according to age groups

A total of 90 patients were assessed for eligibility, and 40 met eligibility criteria, were randomized, and completed the trial; 20 were randomly assigned to each group of treatment (Fig. 1). Baseline clinical and epidemiologic data and parameters in both groups are presented in Table 1. The age varies between 60 and 87 years old (mean 71 years), with 31 (75%) female subjects. Altogether, 28 (70%) had a negative skin prick test. The mean (±SD) PFIN score at baseline was 86,5 ± 35,7 in the saline group and 77,5 ± 27,31 in the corticosteroid plus saline group and measures are shown to decrease progressively as the age group was older. (Fig. 2). The values of VAS and SNOT-22 at baseline were similar in the 2 groups (Table 1).

**Table 1:** Comparative analysis of the patients by baseline demographic and clinical characteristics and scores. PNIF, SNOT-22 and VAS between 2 groups of intervention

Baseline characteristics and parameters	Saline (n = 20)	Corticosteroid plus saline (n = 20)	p-value^a^
**Age, n (%)**			
- 60 a 69	7 (35)	8 (40)	0.609
- 70 a 79	7 (35)	9 (45)	
- ≥ 80	6 (30)	3 (15)	
**Women, n (%)**	14 (70)	17 (85)	0.451
**Result of Prick test, n (%)**			
- Allergic Rhinitis (AR)	6 (30)	6 (30)	
- Nonallergic Rhinitis (NAR)	14 (70)	14 (70)	1.000
**Asthma, n (%)**	2 (10)	3 (15)	1.000
**Smoking, n (%)**	1 (5,0)	0 (0)	1.000
**Eosinophilia, n (%)**	3 (16,7)	5 (27,8)	0.691
**IgE elevated, n (%)**	6 (30)	6 (30)	1.000
**PNIF**^b^	86.5 ± 35.7	77.5 ± 27.31	0.505
**SNOT-22**^b^	36.7 ± 19.8	35.0 ± 17.1	0.920
**VAS**^b^	5.0 ± 1.8	5,8 ± 2,3	0.249

a Chi-squared test.

b Mann-Whitney test (1-tailed)

### Efficacy

The difference in PFIN score between the 2 groups of treatment was not significant at week 2 (79.5 in the corticosteroid plus saline group and 82.0 in the saline group; p = 0.37). In subgroups with increased total IgE or coexisting asthma or allergic rhinitis, the use of mometasone furoate plus saline did not show statistically significant differences in primary and secondary outcomes (Table 2). Also, in nonallergic patients, we could not detect a significant improving in PFIN (72.9 in the corticosteroid plus saline group and 82.1 in saline group; p = 0.688) or reduction of VAS (4.1 in the corticosteroid plus saline group and 5.4 in the saline group; p = 0,056) and SNOT-22 (32.3 in the corticosteroid plus saline group and 24.3 in saline group; p = 0,095) between the 2 groups of treatment. There were no treatment-associated adverse events observed in either group.

**Table 2:** Analysis of primary and secondary outcomes by strata of individuals.

Strata	Outcomes	After intervention		p-value^a^
		Saline spray	Corticosteroid + saline	
		Mean ± SD	Mean ± SD	
All the patients (n=40)	SNOT22	29.2 ± 15.0	28.6 ± 17.8	0.931
	VAS	4.9 ± 2.3	4.1 ± 2.0	0.250
	PNIF	82.0 ± 37.4	79.5 ± 32.0	0.741
Allergic Rhinitis (n=12)	SNOT22	21.8 ± 12.8	38.7 ± 18.1	0.108
	VAS	3.7 ± 2.4	4.0 ± 0.9	0.660
	PNIF	81.7 ± 34.3	95,0 ± 42,3	0.844
Nonallergic Rhinitis (n=28)	SNOT22	32.3 ± 15.2	24.3 ± 16.5	0.189
	VAS	5.4 ± 2.1	4.1 ± 2.4	0.112
	PNIF	82.1 ± 39.8	72.9 ± 25.5	0.688
IgE elevated (n=12)	SNOT22	33,5 ± 13,9	22.8 ± 22.7	0.398
	VAS	5.0 ± 2.3	3.0 ± 0.9	0.076
	PNIF	80.0 ± 34.1	83.3 ± 26.6	1.000
IgE non-elevated(n=28)	SNOT22	27.3 ± 15.6	31.1 ± 15.7	0.489
	VAS	4.8 ± 2.4	4.6 ± 2.2	0.812
	PNIF	82.9 ± 39.7	77.9 ± 34.9	0.740
Asthma (n=5)	SNOT22	43.0 ± 9.9	18.0 ± 22.5	0.300
	VAS	5.5 ± 2.1	2.7 ± 0.6	0.100
	PNIF	65.0 ± 21.2	83.3 ± 41.6	1.000
Without asthma (n=35)	SNOT22	27.6 ± 14.9	30.5 ± 17.0	0.584
	VAS	4.8 ± 2.4	4.4 ± 2.1	0.526
	PNIF	83.9 ± 38.7	78.8 ± 31.6	0.581

a Mann-Whitney test (1-tailed)

## Discussion

To our knowledge, this is the first study that tested the efficacy of adding a corticosteroid nasal spray to nasal saline in improving nasal patency and quality of life and reducing the severity of symptoms in elderly patients with both types of chronic rhinitis.

In this study, the association of topical nasal corticosteroid with saline was not superior to the use of saline alone for the improvement of nasal patency, symptoms of rhinitis and quality of life in the elderly. Furthermore, in subgroups analysis, the addition of corticosteroid did not improve PFIN, VAS for the combined nasal symptoms or the SNOT-22 scale.

The peak nasal inspiratory flow was used in our study for the objective evaluation of the severity of rhinitis by translating the level of nasal obstruction, and any of the present interventions did not influence it. Nasal blockage is a common problem in daily practice, and it interferes substantially in the quality of life of the patients.^3^ There is conflicting data about the correlation between the sensation of nasal obstruction and the objective measure of the nasal flow. Panagou et al. when analyzing 254 patients with rhinitis, asthma or nasal deformity showed that values of PNIF correlated poorly with rhinomanometry and patient-reported measures,^11^ and Morrissey et al. in an observational study with 154 nasal surgery patients found that there was no relation between improvements in nasal subjective scores and PNIF values.^12^ In spite of that, Wilson et al. showed that PNIF values correlated significantly with nasal symptoms in individuals with allergic rhinitis who used nasal corticosteroids^11^ and similarly Teixeira et al. found an association between values of the VAS for nasal obstruction and PNIF measures in 60 healthy volunteers after using a nasal mucosa constrictor.^13^ Measures of PNIF can fluctuate within the same individual caused by daily variations^14^ (higher values at the end of the day), patient cooperation, environmental temperature and influences of the lung function.^15^

Additionally, the test can be difficult for those who have nasal valve collapse at inspiration.^16^ As suggested by Panagou et al., the sensation of nasal obstruction can be a consequence of changes in the temperature in the nasal cavity, more than nasal blockage per se.^11^ The weak correlation between self-reported nasal obstruction scores and objective measure of nasal patency in the elderly shows that probably the PNIF is not as accurate in this age group as in young people.

In our study, PNIF values decrease with age. In a cohort of 113 volunteers with ages ranging from 65 to 84 years, Ottaviano et al. observed a general PNIF values diminution with age and proposed that the progressive cartilage weakening causes alar collapse and a tip of the nose drop, producing nasal obstruction and a gradual reduction in nasal patency.^17^

There were 30% of the participants in the study who had positive prick test, but the addition of mometasone furoate did better in nonatopic patients. This finding can be explained by the fact that rhinitis in older adults rarely is purely allergic^5^ (even in those who have positive skin prick test there is some significant nonallergic component) and the prevalence of allergy tends to reduce with ageing. This phenomenon can be attributed to immunosenescence.^18^ In our study, 30% of the patients had elevated levels of total IgE and half of them are also atopic. The decline in the immune function observed in older patients could be a result of the decrease in levels of total and specific IgE. Mediaty et al. showed in a retrospective study with more than 500 individuals with several atopic disorders that levels of total IgE were lower in older patients who had allergic rhinitis or asthma.^19^

Nonetheless, in a prospective study, Di Lorenzo et al. followed patients with allergic rhinitis for 15 years and found no change in levels of total IgE with time. In the same period, it was showed a reduction in the levels of specific IgE. As pointed out by the author, it seems that the immunosenescence affects only specific immune responses and the sensitization to particular allergens (e.g., skin prick test). The total IgE is an unspecific marker of atopy and levels keep stable along of the time.^20^

The association between asthma and allergic rhinitis is prevalent. Up to 90% of patients with asthma also have allergic rhinitis.^21^ A non-specific bronchial hyperreactivity is elevated in those who have allergic rhinitis.^22^ In a retrospective study with almost 5,000 patients with asthma and allergic rhinitis, Crystal-Peters et al. noted that events related to asthma had significantly reduced with the use of nasal topic corticosteroid for the nasal problem.^23^ In our study, 20% of the individuals with asthma and 50% of those with elevated total IgE had a positive skin prick test.

There is no sole treatment for nonallergic rhinitis because its etiology is diverse, but the control of symptoms is always the final goal.^24^ However, the same drugs used in allergic rhinitis in the elderly are also prescribed in nonallergic rhinitis in this age group, because of the lack of scientific evidence.^25^ Furthermore, there is little information even for the treatment of allergic rhinitis in older patients. In one of the few studies in this field, Grossman et al. revealed that elderly patients with allergic rhinitis had clinical improvement through the TNSS score (total nasal symptoms score) in 15 days using mometasone furoate without significant side effects.^26^ On the other hand, the use of isotonic saline for nasal irrigation is the treatment of choice for cleaning the thick mucus and clusters and reducing the sensation of nasal dryness.^26^ That is because the humidification of the nasal cavity is a practical aim since the atrophy of the nasal mucosa with ageing leads to an increase in nasal space and, paradoxically, to nasal blockage.^27^ Randomized trials suggest that the use of saline, no matter the technique to be used, is a safe, well-tolerated and effective method for this purpose.^28^ As in the literature, there were no reports of side effects in our study, both with mometasone furoate and isotonic saline spray.

The limitations of the study are the small sample size, the open-label intervention and the short time of treatment and follow-up. It was not possible to analyze the effect of subjects with mixed rhinitis (rhinitis not purely allergic) separately, and it could be considered another limitation of the study.

## Conclusions

In conclusion, there was no significant benefit of adding corticosteroid to a saline regimen on the improvement of nasal patency, the severity of symptoms, and quality of life among elderly with rhinitis in the study population. The two regimens had a similar safety profile.

## Ethics approval and consent to participate

The ethic committee of Hospital Univesitário Antônio Pedro/Universidade Federal Fluminense approved this study (reference number: 2.467.671).

## Consent for publication

Patients signed an informed consent document to participate in the study.

## Availability of data and materials

All the clinical and laboratory data are available, on request, at torcortes@yahoo.com.br out of respect for the patients' anonymity.

## Competing interests

The authors declare that they have no competing interests.

## Funding

The authors declare that no funding was received for the present study.

## Authors' contributions

VC, BO, JRM and JLB designed of and conceived the study. VC and JLB recruited the patients. VC, BO and JLB performed the study. VC, JRM and JLB analysed the data. VC, BO and JLB wrote the manuscript. VC, BO, JRM and JLB revised the manuscript. All authors read and approved the final manuscript.
